# Re-examining Vaccine Staggering Within Hesitancy Frameworks

**DOI:** 10.3389/fimmu.2021.662814

**Published:** 2021-05-24

**Authors:** Ana Santos Rutschman, Timothy L. Wiemken

**Affiliations:** ^1^ Saint Louis University School of Law, Center for Health Law Studies, Saint Louis University, Saint Louis, MO, United States; ^2^ Institute for Vaccine Science and Policy, Saint Louis University, Saint Louis, MO, United States; ^3^ Saint Louis University School of Medicine, Department of Internal Medicine, Division of Infectious Diseases, Allergy, and Immunology, Saint Louis University, Saint Louis, MO, United States; ^4^ Systems Infection Prevention Center, Saint Louis University, Saint Louis, MO, United States

**Keywords:** vaccine, immunization, hesitancy, anti-vaccination, infrastructure

## Introduction

Although vaccines are highly regulated and among the most cost-effective and successful public health interventions for preventing infectious diseases, vaccine confidence across the United States is declining (1). Vaccine confidence accounts for behaviors that show “acceptance of vaccines administered at the recommended times” (2). Part of the recent decline in vaccine acceptance is linked to individuals defined as vaccine hesitant, an expression signaling a behavioral departure from adherence to vaccine schedules. In 2019, the World Health Organization (WHO) added vaccine hesitancy to the list of leading global public health threats, though current definitions of vaccine hesitancy lack specificity leading to operational difficulties for intervention. Formal definitions encompass disparate phenomena, ranging from delays in vaccine timelines (i.e. staggering/alternative schedules) to vaccine refusal (3). In this perspective, we focus on the case of vaccine staggering as it relates to the larger issue of departures from well-established vaccination schedules. Vaccine staggering or alternative vaccine scheduling is the practice of reducing the number of vaccines given at a single time point or within a specified time period. Here, we identify three problems with the absorption of vaccine staggering into hesitancy frameworks as currently defined by the World Health Organization. Further, we argue that the lack of separate treatment of vaccine staggering is likely to contribute not only to conflicting discourses on hesitancy-related topics in general but also detrimentally affect interventions to deal with hesitancy at large. We identify there three main domains in which to focus to begin remedying these issues: conceptual, informational, and infrastructural.

## Discussion

### Issue 1: Conceptual

Although the WHO provides a definition of vaccine hesitancy (“delay in acceptance or refusal of vaccines despite availability of vaccination services.” (3)), it is not functional from the standpoint of public health intervention. Per the current definition, vaccine staggering can be subsumed into “delay in acceptance” frameworks. However, the actual practice of staggering is materially different from other forms of delaying vaccination, which include a broad set of heterogeneously motivated behaviors, ranging from delays attributable to philosophical or religious convictions to delays linked to informational deficits or hardship (3, 4). By integrating staggering into the broad and amorphously defined category of “hesitancy,” current frameworks fail to account for materially meaningful differences between delays resulting from staggering practices—which if properly defined result in vaccination occurring at a later date from the range recommended in applicable vaccination schedules—and behaviors resulting in much more protracted temporal delays, which may ultimately result in failure to receive a vaccine.

The conceptual problem identified above has further implications for research on staggering and hesitancy alike. Ongoing research on different aspects of vaccine hesitancy has traditionally been predominantly focused on parental behavior, although some strands of literature have recently turned to issues related to adult hesitancy. As a field of research in need of dedicated analytical and empirical attention, vaccine staggering practices should be investigated both within the context of parental and non-parental behaviors.

### Issue 2: Informational

From a clinical standpoint, the relationship between immunity and temporally concentrated administration of vaccine in both adult and pediatric populations remain understudied. Although reports from the laboratory suggest altered immune response with fewer concurrent vaccines provided (5), this may not translate to clinical practice where coadministration of at least some vaccines has not shown any decreased immune response (6). Consequently, the possible utility of vaccine staggering, or lack thereof, is not properly understood. However, it cannot be underscored enough that if there is only a single chance to give/receive all scheduled vaccines, this opportunity must be utilized regardless of the theoretical potential for an altered (either enhanced or reduced) immune response. Again, there is no evidence of any harm from provision of multiple scheduled vaccines concurrently. There are, however, arguments for vaccine timeliness, which is a critical aspect of infection prevention. Unfortunately, definitions of timeliness are variable and may drive confusion in this area (7). Further, refusal in vaccine provision increases infection risk, as we have seen with measles and pertussis over the past decade (8). Despite this knowledge, these arguments outline potential areas of confusion with respect to staggering and showcase the limited study in this area which may drive underinformed or misinformed decision making.

The informational problem is part of systemic deficits in knowledge surrounding vaccine hesitancy. Currently, most vaccine provision and uptake information are concentrated on pediatric populations. We lack reliable data on vaccine provision and uptake in adults, largely due to the lack of federal regulation and national data sources reducing the ability to perform epidemiological studies. In particular, we are missing data on groups at low risk of infection due to vaccine preventable diseases (9). Low-risk groups constitute the majority of adults in the United States, vaccine provision is often limited to when travel vaccines are being administered, or when healthy, non-hesitant individuals have healthcare contact where a vaccine is recommended. In the context of vaccine staggering, even within pediatric populations, there is little beyond laboratory data and virtually no clinical data on the outcomes associated with vaccine staggering. If these gaps in information persist, interventions to curb vaccine hesitancy are likely to be less successful. This problem is further compounded by the continued spread of vaccine misinformation and disinformation through online and offline networks (10).

### Issue 3: Infrastructural

As previously alluded to, there is no national immunization information system. Improvements cannot be made without a thorough understanding of baseline rates of acceptance and therefore proposed interventions to curb vaccine hesitancy are less likely to be successful. This is a difficult area necessitating various stakeholders working to fund, develop and coordinate programs required to move data from a multitude of electronic health record systems across public, private, and outpatient areas providing immunization services. Currently, there are no incentives for stakeholders to link these largely disconnected providers.

## Proposal

We suggest a three-stage approach to address the problems described above. First, public health-oriented organizations should work in a concerted way to provide guidance at both national and international levels. These organizations should explore and clarify the material differences between vaccine staggering and other types of behaviors currently treated under the umbrella of “vaccine hesitancy.” Materially, a request to delay vaccines based on a belief that staggering may be beneficial (and therefore would continue to seek all necessary vaccines after a personally defined sufficient time period has elapsed between vaccines) should not be grouped with delays attributable to other factors, such as distrust or concerns over potential side effects. Moreover, staggering as a form of vaccine hesitancy is so distinct from vaccine refusal that these two behaviors should not be addressed under the same label (hesitancy). Providing conceptual clarification will, in turn, inform scientific, policy and non-technical debates on themes related to vaccine hesitancy. The WHO, particularly through the Strategic Advisory Group of Experts on Immunization, is well placed to do this.

Second, clinical research on the impact of concurrent vaccine provision should be considered a utility, and funding should be earmarked for this purpose. Moreover, we also need funding directed at exploring and providing a better understanding of the reasons that lead different types of populations to stagger vaccine. While immediate interventions from policy-making and public-health oriented institutions may contribute to greater conceptual clarity, the informational status quo will still lack the needed granularity that would, at a later stage, enable these organizations to take a stance in against vaccine staggering or improve current vaccine schedules with real-world data. In short, evidence is necessary to inform policy.

Third, building the appropriate infrastructure for a national immunization system is of utmost importance. This includes building and maintaining a national immunization information system, developing policies mandating reporting of vaccine provision in pediatric and adult populations, developing the programming necessary to link disparate health record systems, and ensuring quality and transparency of said data. Direction of these systems must be a collaborative effort between federal public health officials as well as health record suppliers to ensure data are shared, systems are compatible, and implementation is subsidized or engulfed within national budgets. While the third stage of the proposed approach is of a transversal nature, current informational deficits surrounding vaccine staggering cannot be properly addressed in a siloed fashion. Systemic changes at the infrastructural level are critical to address localized issues, as is the case with vaccine staggering.

## Author Contributions

All authors contributed to the article and approved the submitted version.

## Conflict of Interest

The authors declare that the research was conducted in the absence of any commercial or financial relationships that could be construed as a potential conflict of interest.
